# Stereological and ultrastructural quantification of the afferent synaptome of individual neurons

**DOI:** 10.1007/s00429-013-0523-9

**Published:** 2013-03-12

**Authors:** Pablo Henny, Matthew T. C. Brown, Benjamin R. Micklem, Peter J. Magill, J. Paul Bolam

**Affiliations:** 1 MRC Anatomical Neuropharmacology Unit, Department of Pharmacology, University of Oxford, Mansfield Road, Oxford, OX1 3TH UK; 2 Laboratory of Neuroanatomy, Departamento de Anatomía Normal, Escuela de Medicina, Pontificia Universidad Católica de Chile, Santiago, Chile; 3 Centro Interdisciplinario de Neurociencia, Pontificia Universidad Católica de Chile, Santiago, Chile; 4 Oxford Parkinson’s Disease Centre, University of Oxford, Oxford, UK; 5 Present Address: Department of Bioengineering, Imperial College, London, UK

**Keywords:** Stereology, Juxtacellular, Electron microscopy, Single cell, Neuronal digital reconstruction

## Abstract

Determining the number and placement of synaptic inputs along the distinct plasma membrane domains of neurons is essential for explaining the basis of neuronal activity and function. We detail a strategy that combines juxtacellular labeling, neuronal reconstructions and stereological sampling of inputs at the ultrastructural level to define key elements of the afferent ‘synaptome’ of a given neuron. This approach provides unbiased estimates of the total number and somato-dendritic distribution of synapses made with individual neurons. These organizational properties can be related to the activity of the same neurons previously recorded in vivo, for direct structure–function correlations at the single-cell level. The approach also provides the quantitative data required to develop biologically realistic models that simulate and predict neuronal activity and function.

**Electronic supplementary material:**

The online version of this article (doi:10.1007/s00429-013-0523-9) contains supplementary material, which is available to authorized users.

## Introduction

As exemplified by early neuroanatomical studies, ex vivo observation of the shape and connectivity of single neurons can provide enduring insights into neuronal function (Cajal 1899). In spite of the advances in neuroanatomy during the last century, there remains a need for approaches that can provide detailed and quantitative descriptions of neuronal structure, connectivity, and structural–functional correlations at the individual cell level (Pinault 1996; Swanson 2007; Javier and Kreitzer 2012; Spruston 2008; Klausberger and Somogyi 2008; DeFelipe 2010). These descriptions are necessary for revealing the mechanisms underlying the activity of neurons, circuits and brain, and for the generation of computational models for simulation of single neuron and network activities (Grillner et al. 1995; Jarsky et al. 2005; Izhikevich and Edelman 2008; Katz et al. 2009).

Methods used to quantify the structure of neurons and circuits, as characterized by highly diverse somatic, dendritic and axonal arrangements, should be based on random sampling and unbiased counting, as provided by stereology (Coggeshall and Lekan 1996; Saper 1996; Howard and Reed 1998; West 1999; Avendano 2006; Vanhecke et al. 2007). Also, demonstration of synaptic connectivity should, ideally, be based on ultrastructure (DeFelipe 2010). We introduce a protocol to accurately estimate the synaptic inputs of an individual neuron using stereological principles: synapses are randomly and systematically sampled and counting is carried out at the electron microscopic level using a physical fractionator (Howard and Reed 1998; West 1999; Tang et al. 2001; Sterio 1984). Sampled synapses are mapped onto previous light-microscopic reconstructions of the same neurons (Glaser and Glaser 1990; Ascoli 2006) to examine the distribution and density of inputs within different somato-dendritic compartments, thus defining key features of the neuron’s afferent ‘synaptome’ (DeFelipe 2010). These data can be then correlated with activity profiles, as identified from the previous in vivo electrophysiological recordings of the same neurons (Pinault 1996; Duque and Zaborszky 2006; Klausberger and Somogyi 2008; Henny et al. 2012; Javier and Kreitzer 2012).

## Materials and methods

### Neuron labeling and processing of tissue (Supplementary Methods I)

**Fig. 1:**
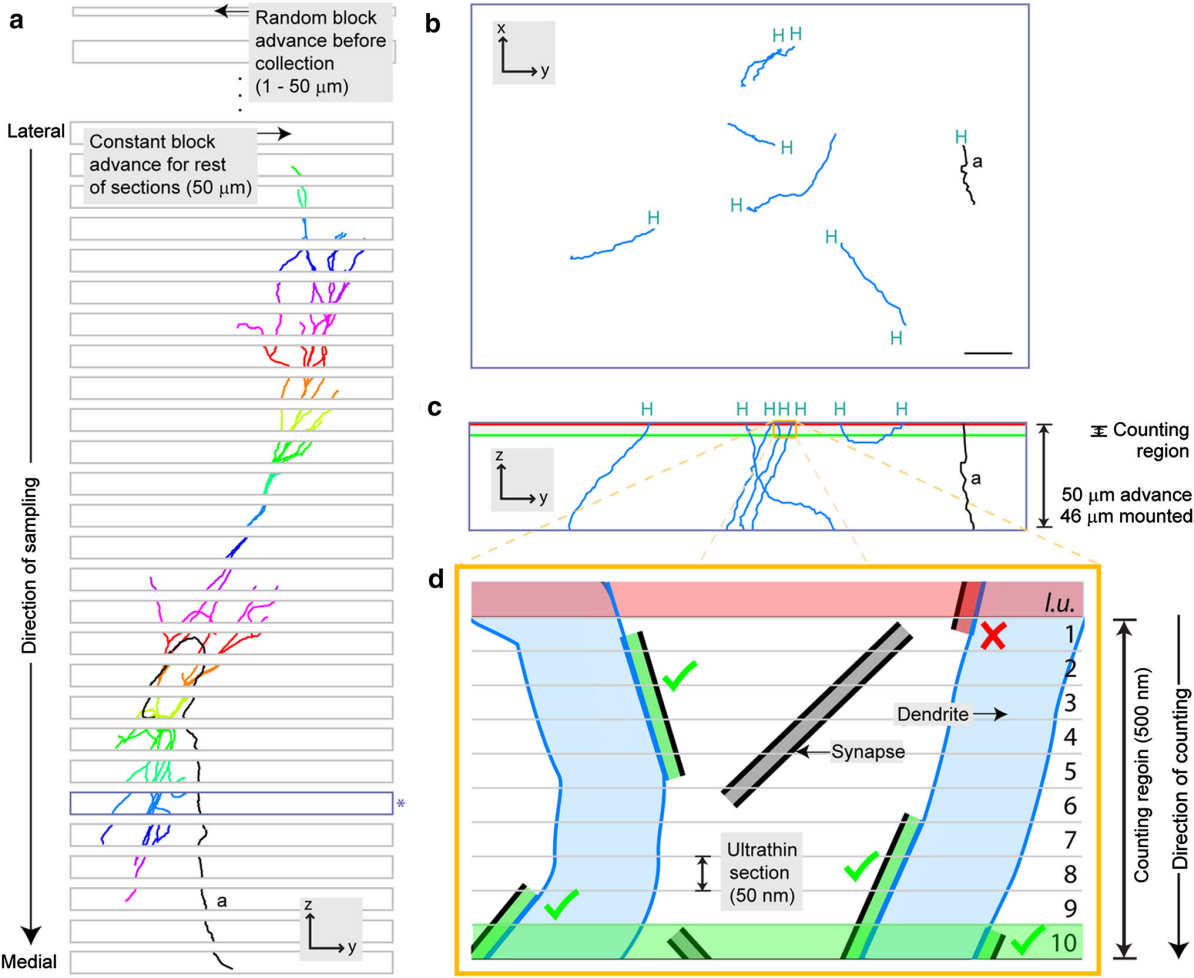
Overview of sampling and synapse counting procedures. **a** The brain is sectioned in the parasagittal plane, lateral to medial. Before collection, the cutting stage is advanced a random distance (1–50 μm). The dendrites of the labeled neuron are shown in *different colors* in each section. Synapses formed with dendrites are counted in a counting region of fixed height located at the top surface of each section (shown in **c** and **d** for *blue-outlined* section marked with the *asterisk*). The axon (*a*) is shown in *black*. **b** Enlarged view of *blue-outlined* section in (**a**) rotated orthogonally to show dendritic and axonal (*a*) fragments that were digitally traced at high magnification. Fragments ending at the top surface of the section (‘high endings’; H) correspond to the location of the counting region. **c** Enlarged view of *blue-outlined* section in (**a**), (same orientation to (**a**), but orthogonal to **b**). Putative synapses (not shown) made with the high endings (H) of dendritic fragments are counted within a counting region of 500 nm height, on the top of the 46 μm-thick mounted section. **d** *Orange box* in **c**, expanded for two dendrites, with a representation of the procedure for counting of synapses on dendritic fragments. The counting region consists of ten ultrathin serial sections (numbered 1–10), each cut with a block advance of 50 nm. A look up (*l.u.*) section (Sterio 1984; Howard and Reed 1998) is located above the counting region (*red shade*). Synapses in *gray* represent those not formed with labeled dendrites. Synapses in *green* represent those formed with labeled dendrites that are counted (*green ticks*) according to stereological counting rules. Synapse in *red* is formed with a labeled dendrite but is not counted (*red cross*) because its top is not within the counting region (see “Materials and methods”)

The labeling of neurons was carried out in vivo (Pinault 1996; Brown et al. 2009; Henny et al. 2012). After fixation, the brain was serially sectioned on a vibrating microtome using a constant block advance (or “thickness”, here 50 μm). Random sampling was ensured by advancing the cutting stage a random distance (between 1 and 50 μm), before sections were collected (Coggeshall and Lekan 1996; Howard and Reed 1998) (Fig. 1a). After optional neurochemical characterization of the neuron and/or presynaptic terminals (Henny et al. 2012) (Supplementary Methods I), the entire neuron was revealed for digital reconstruction of the neuron and ultrastructural analysis of its synaptic inputs (Bolam 1992).

### Digital reconstruction, tissue re-embedding and stereological counting (Supplementary Methods II)

**Fig. 2:**
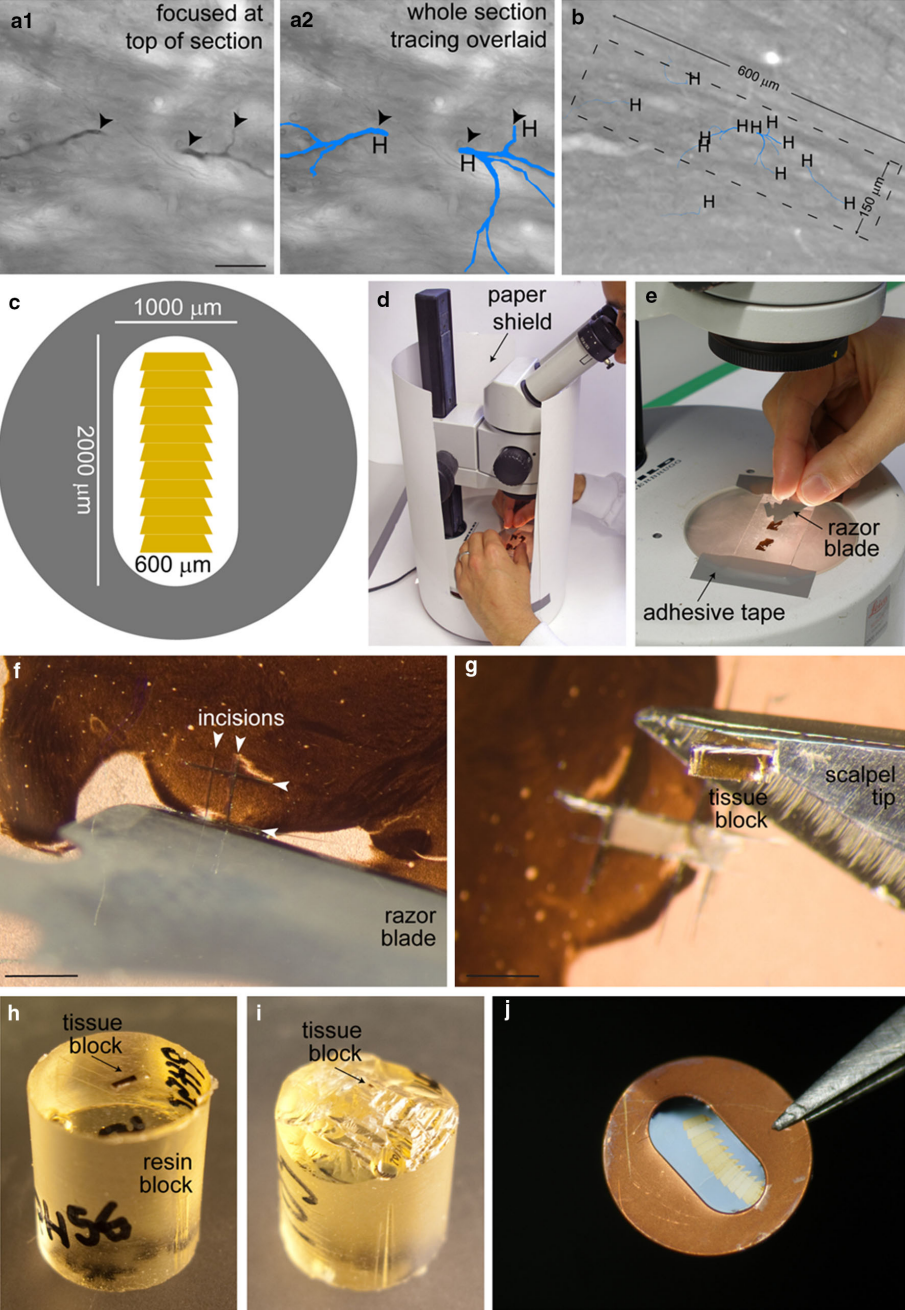
Re-embedding and ultrathin sectioning. **a**, **b** Acquisition of images of dendritic fragments for correlated light and electron microscopy. **a** Pair of high magnification images of peroxidase (neurobiotin)-labeled dendritic fragments at their high endings (*arrowheads*), with (**a2**) and without (**a1**) the digital tracings overlaying them (*blue*). All dendritic high endings are visited and imaged, and marked (H) on the digital reconstruction (**a2**), before the re-embedding process starts. **b** Low-magnification image of the same dendritic fragments in tissue that will be re-embedded, trimmed down, and sectioned on the ultramicrotome for electron microscopic analysis. A *dashed rectangle* is overlaid to show the desired approximate final size and orientation of the trimmed (**i**) tissue block. Note that not all dendritic high endings (H) in a given section are necessarily captured in the same tissue block (*dashed rectangle*). **c** A representation of a single-slot, pioloform-coated electron microscope grid holding a series of ultrathin sections, showing the dimensions (150 μm × 600 μm) of the trimmed tissue block (**i**) that will allow series of more than 10 ultrathin sections to be collected. **d**–**i** Key steps in isolation, re-embedding and trimming of tissue blocks. **d** To minimize the risk of losing the tiny tissue block during the excision and handling of it, a clean white lab coat is worn by the researcher, and a paper shield is placed around the dissection microscope. **e** The microscope slide is secured to the dissection microscope to facilitate cutting. **f** Incisions flanking the region of interest (*arrowheads*) are made to release the tissue block. **g** The tissue block is freed with a fine scalpel blade. **h** The bottom surface of the tissue block is glued to the resin block. **i** The tissue block is trimmed to achieve the desired dimensions (approximately 150 μm × 600 μm). **j** Ultrathin sections are collected as a ribbon on the single-slot, pioloform-coated grids. The ribbon shown has about 12 ultrathin sections. *Scale bars,* **a1**, 20 μm (applies also to **a2**); **f** and **g**, 1 mm

The cell body, dendrites and axon of the labeled neuron were traced at high magnification using vector-based tracing software (Neurolucida™, MBF Bioscience) (Glaser and Glaser 1990; Ascoli 2006) (Figs. 1a, b, 2a, 5a). One file containing the digital reconstruction was left unspliced and used to carry out the sampling and counting steps (Fig. 1; see also Supplementary Methods II). A second file was spliced to provide quantitative data about somato-dendritic architecture (Fig. 5b) and distribution of synapses (Fig. 5c, d).

During tracing, fragments of dendrites and cell body located at the top surface of each 50 μm-thick section (46 μm when dehydrated and mounted in resin) were identified and imaged (Figs. 1b, 2a, b). Single or groups of dendrites or cell fragments were excised from the microscope slide, re-embedded, trimmed and re-sectioned on an ultramicrotome (Fig. 2c–j) in series of ultrathin sections using a block advance of 50 nm. Series for all fragments were collected. This allowed to define a counting region at the surface of all sections, formed by ten 50 nm ultrathin sections (500 nm), plus one look up ultrathin section at the top of the series (Sterio 1984) (Fig. 1c, d). This procedure was systematically applied to the tissue every 50 μm, i.e. to each original section containing a labeled fragment (Fig. 1a).

**Fig. 3:**
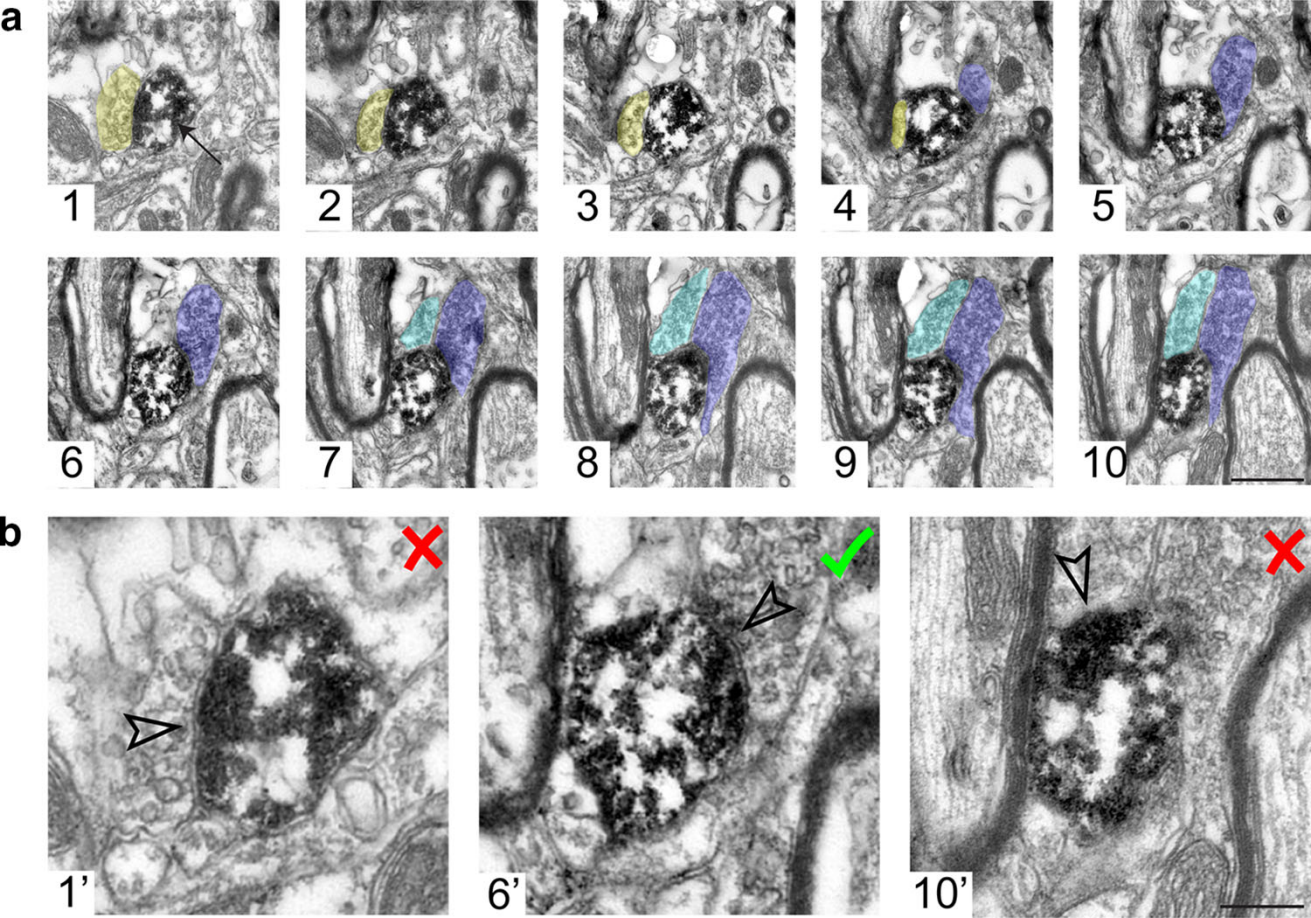
Counting of synapses through series of ultrathin sections. **a** Micrographs of a high ending of a neurobiotin-labeled dendritic fragment (as revealed by the peroxidase reaction; *arrow* in *1*) in a series of ten 50 nm-thick ultrathin sections, numbered from most superficial (*1*) section onwards. For clarity, three different axon profiles are colored; *yellow* (*1–4*), *dark blue* (*4–10*) and *light blue* (*7–10*), and only those sections forming the 500 nm counting region are shown (see Fig. 1d). **b** Following the ‘only tops’ rule to ensure unbiased sampling, the synapse established by the *yellow* terminal (in *1′*, corresponding to 1 in **a**) is not counted because its ‘top’ is not within the counting region (similar to *red* synapse in Fig. 1d). The synapse established by the *dark blue* terminal (in *6′*, corresponding to 6 in **a**) is counted because its top is within the counting region. A profile (*light blue*,* 7*–*10*) that cannot be unequivocally identified as forming a synapse with the labeled dendrite (such as in *10′*, corresponding to *10* in **a**) within the counting region is not counted. *Scale bar* **a** 500 nm in micrograph *10* applies to all images; **b** 200 nm in image *10′* applies to all three images

All labeled dendritic or cell fragments were identified in the electron microscope and micrographs were taken sequentially through series of 50 nm ultrathin sections (Fig. 3). The series of images were opened off-line and synapses counted using a fractionator probe (the optical fractionator) with stereological analysis software (Stereo Investigator™, MBF Bioscience). Synapses made with labeled profiles were counted only once through the series and only if their tops were present within the 500 nm counting region (the ‘top rule’; see Figs. 1d, 3) (Howard and Reed 1998; West 1999).

After repeating the counting procedure for all dendritic fragments, the total number of counted synapses was obtained. This value was then multiplied by the reciprocal of the fraction of tissue effectively sampled (called the height sampling fraction) to obtain an estimate of the total number of synapses:

Total number of synapses = counted synapses × (height sampling fraction)^−1^

with

Height sampling fraction = counting region height × (section thickness after mounting)^−1^

In our case, counting region height = 500 nm, and section thickness after processing = 46 μm (Table 1; Fig. 1c, d). Values for coefficient of errors [CE, a measure of the accuracy of the estimates (West et al. 1991; Howard and Reed 1998)] associated with the sampling strategies could be also obtained from the stereological software.

**Table 1:** Example of values obtained after analysis of a single neuron

Item	Value
*In relation to sampling procedure*	
Number of tissue sections analysed	25
Number of dendritic fragment high endings	60
Percentage dendritic fragment high endings analysed	100
Percentage of fragments with synapses	73
*After correlated light and electron microscopy*	
Synapes counted	114
Estimation of total number of synapses^a^	10,488
Gundersen CE^b^	0.09
Cruz-Orive CE^b^	0.11
Approximate total dendritic length (μm)^c^	7,076
Approximate total somatodendritic surface area (μm^2^)^c^	16,250
Approximate linear density (synapses/μm)	1.48
Approximate surface area density (synapses/μm^2^)	0.65

^a^Estimation of synapses = counted profiles/height sampling factor = 114/0.01087 (with height sampling factor = counting region height/mounted thickness = 0.5/46 μm = 0.01087, see text for details)

^b^ *CE* coefficient of error, as provided by the optical fractionator probe. It corresponds to the coefficient of variation of the sampling distribution. Values reflect the precision of stereological estimates. See Howard and Reed (1998) and http://www.stereology.info/coefficient-of-error/

^c^Values for length and area as obtained from tracings in Neurolucida with 100×/1.4 NA objective (see text for details)

**Fig. 4:**
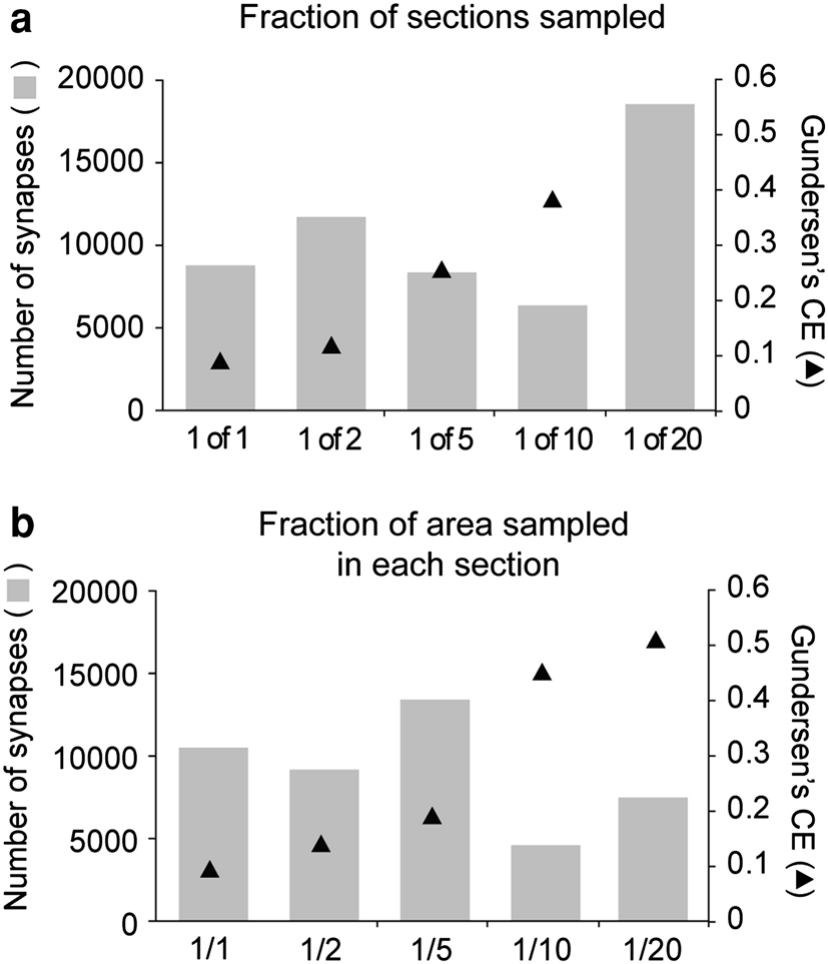
Alternative sampling schemes with reduced number of sections or reduced area per section sampled. **a** The estimated number of synapses (*gray bars, left Y axis*) and Gundersen’s CE values (*triangles, right Y axis*) obtained with reduced numbers of sections sampled (*X axis*). A single probe was run for each scheme, for which a single, randomly selected starting section was chosen for the 1 of 2 (1st section), 1 of 5 (5th section), 1 of 10 (1st section) or 1 of 20 (5th section), except for the 1 of 1 regime, where values correspond to those obtained after the full protocol is applied (see Table 1). The CE value for the 1 of 20 scheme was not calculated as there were only 2 sections (5th and 25th section) sampled. **b** The estimated number of synapses and CE values obtained with reduced sampled areas in each section (*symbols* and *axes* as in **a**). A counting frame of 100 × 100 μm (10,000 μm^2^) was used with sampling grids of increasing size (141 × 141 μm (~20,000 μm^2^), 224 × 224 μm (~50,000 μm^2^), 316 × 316 μm (~100,000 μm^2^) and 447 × 447 μm (or ~200,000 μm^2^) for ~1/2, ~1/5, ~1/10 and ~1/20 of the area in each section, respectively) and for which a randomly selected starting point was chosen each time

In this protocol, all fragments located at the top of a tissue section were sampled (Fig. 1a, b). This was done using a counting frame (the probe that is systematically spaced over the *XY* plane) of the same size as the sampling grid [the lattice upon which counting frames are systematically spaced; in this case, 100 × 100 μm (or 10,000 μm^2^)]. Alternative sampling strategies were examined by reducing the area sampled in the *XY* plane (using a counting frame smaller than the sampling grid), or by reducing the number of sections sampled (Fig. 4 and Supplementary Methods II).

### Synaptic distribution and density (Supplementary Methods II)

**Fig. 5:**
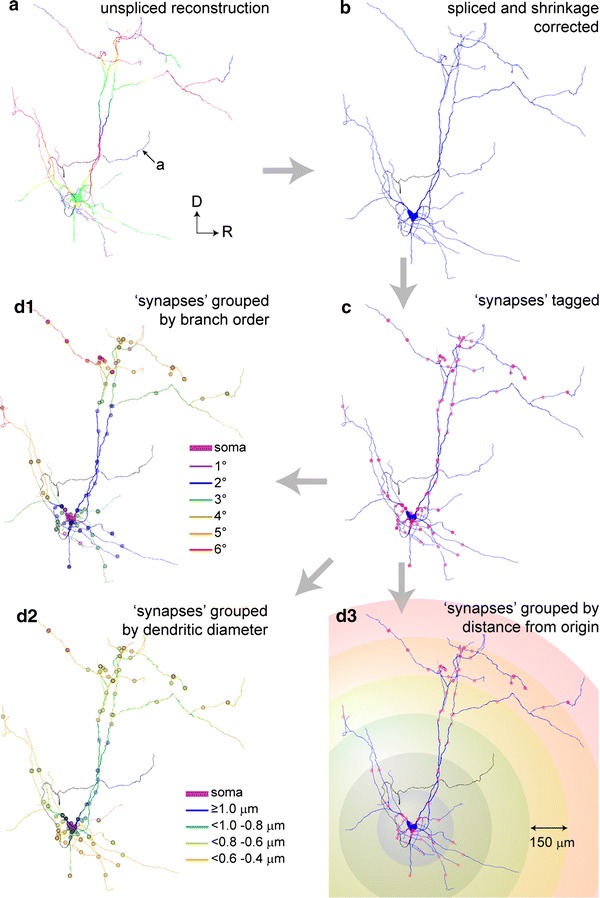
Mapping synapse distributions onto reconstructed neurons. **a** Unspliced digital reconstruction of the same neuron as in Fig. 1a (oriented as in Fig. 1b, *same color* and *legend code* for fragments) after all fragments of dendrite and axon have been traced but before they are spliced together. **b** Spliced reconstruction of the same neuron (including correction for tissue shrinkage). Connectivity, location, and estimates of the length and surface area of all dendritic segments can be extracted using a dedicated software (see “Materials and methods”). **c** Reconstruction after counted synapses are tagged (*red dots*). Combining stereological and reconstruction data thus allows the afferent connections of the individual neuron to be precisely mapped. **d** Number and density of synapses can be studied as a function of dendritic branch order (**d1**, dendritic segments and synapses *color coded* by branch order), mean dendritic diameter (**d2**, dendritic segments and synapses *color coded* by grouped segment diameters) or distance from the soma (**d3**, as located in concentric shells of increasing ratio, using a Sholl analysis). Values of length or surface area are used to obtain approximate measures of synaptic density (see text and Fig. 6)

**Fig. 6:**
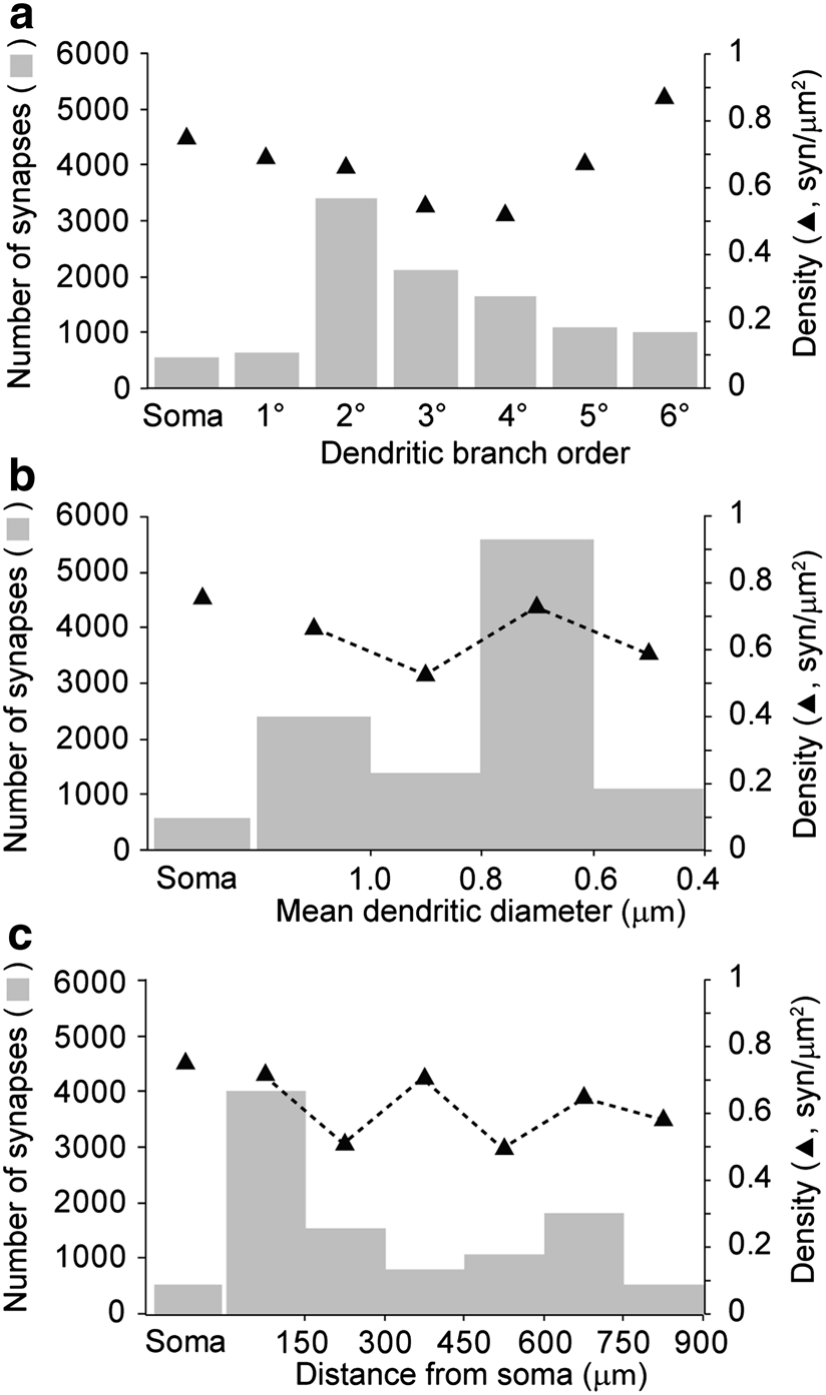
Examples of distribution, number and density of synapses made with a single neuron (**a**–**c**). Estimated numbers of synapses (*gray bars, left Y axis*) and approximate surface-area densities of synapses (*triangles, right Y axis*) as a function of the location of synapses at the soma or at dendritic segments of increasing branch order (**a**), increasing mean diameter (**b**) or increasing distance from soma, as using concentric shells of increasing ratio (150 μm) defined by Sholl analysis

Stereological counts were integrated with the data from the digital reconstructions of the same neurons to map the distribution of synapses in relation to the dendritic branch order, dendritic caliber or distance from the cell body (Figs. 5, 6). The procedure involved digitally ‘tagging’ each of the counted synapses at their precise locations on the reconstructed dendrites and soma (Fig. 5a–c). Values for approximate dendritic length and surface area in the digital reconstruction were used to approximate synaptic density of specific dendritic compartments (Fig. 6).

## Results

The neuron shown in this article was estimated to receive 10,488 synapses, with a Gundersen’s CE of 0.09 (Table 1). To examine the relation of CE values or synaptic number estimations with the sampling procedure, sampling schemes with reduced numbers of sections (1 of 2, 5, 10 or 20; Fig. 4a) or reduced *XY* areas sampled in each of the sections (~1/2, ~1/5, ~1/10, ~1/20 of area; Fig. 4b) were tested. Reduced sampling was associated with more variable estimates and larger CE values, indicative of less accurate estimates as sampling decreases (Howard and Reed 1998; West 1999). Synapses tended to aggregate in low (2nd–3rd) order (Fig. 6a, left axis), middle (0.6–0.8 μm) diameter (Fig. 6b, left axis) or close (<150 μm) to the soma (Fig. 6c, left axis) dendrites. Synaptic density (maximal at soma and high (6th) order dendrites, right axis) and number (left axis) showed a reciprocal distribution as a function of branch order (Fig. 6a). These differences in distributions were not apparent as a function of dendritic diameter or distance to the soma (Fig. 6b, c).

## Discussion

A critical issue about neuronal function is the mechanism by which individual neurons ‘weigh up’ and integrate the synaptic inputs that act on their membrane domains (Spruston 2008). Because the influence of synaptic inputs depends on their number and location made with individual neurons (Hausser et al. 2000; Segev and London 2000; Jarsky et al. 2005; Spruston 2008; Katz et al. 2009; Petreanu et al. 2009), revealing the mechanisms of synaptic integration requires accurate data about number and localization of synaptic inputs across the somato-dendritic domain.

The likelihood of any group of synapses being sampled during the stereological procedure is proportional to their absolute number of synapses, and not to their location, shape or size. Thus, this protocol provides estimates that tend toward the true number (Coggeshall and Lekan 1996; Howard and Reed 1998; West 1999). As stereology-based estimates of the synaptic number of single neurons (Henny et al. 2012) have not been performed before, the results cannot be directly compared to independent published data. However, evidence supports the method provides accurate values. First, the CE values (being a measure of the accuracy of the estimates (West et al. 1991)) for the sampling scheme (CE = 0.09) (Table 1), or for those schemes obtained using half of the number of sections (CE = 0.12) or area in each section (CE = 0.14) (Fig. 4), lie within values previously reported for stereological analyses (Tang et al. 2001; Gritti et al. 2006; Faunes et al. 2012). Second, our estimates for a single neuron (~10,500 synapses) or the mean of six neurons (~8,000 synapses on average (Henny et al. 2012)) are of a similar magnitude to synapse-to-neuron ratios obtained in other brain regions using stereological approaches (~11,000 synapses per neuron in layers II–III of rat visual cortex (Miki et al. 1997); ~7,200 synapses (Tang et al. 2001) per neuron (Pakkenberg and Gundersen 1997) in human neocortex). Finally, estimates are within the range of synapse-to-neuron ratios obtained using values of synaptic density from sets of cell compartments of individual neurons [~32,000 for pyramidal cells (Megias et al. 2001) and ~2,200–16,000 for interneurons of the rat hippocampus (Gulyas et al. 1999)]. Technical developments that will allow a more direct comparison with our data include protocols that optimize the time of tissue processing and sampling strategies, such as automated ultrastructural reconstructions, improved ultrastructural stereological quantifications and light microscopy-based identification of putative synapses (Denk and Horstmann 2004; Vanhecke et al. 2007) (see below).

Random sampling of synapses across the entire neuron avoids focusing a priori on any specific compartment of the neuron. Because in our protocol only a small fraction of the tissue is sampled, single neuronal compartments such as the cell body, the axon initial segment or any specific type of dendrite (e.g. the thickest, thinnest, most distal, etc.) may not always be sampled, as they may not locate at the top of the section (i.e. within the counting region) in every neuron. Thus, the protocol admittedly may not have enough resolution to provide precise estimates of synaptic number or density in these structures. The exclusion of single compartments due to random sampling, however, should not be taken as affecting the accuracy of the estimates for total synaptic number *per se*; rather, it is a consequence of an unbiased design (Howard and Reed 1998; West 1999). Protocols with larger sampling fractions or specifically targeting these compartments could provide better resolution. On the other hand, synapses on compartments present throughout the dendritic domain of some neurons, such as dendritic spines (including spine’s heads or necks) should, according to an unbiased design, be sampled proportional to its presence throughout the neuron. Therefore, their number should not be under or over-estimated.

In contrast to the number and distribution of synapses, values for length and surface area are not unbiased because they are based on the representation of dendrites as simplified tubular structures (Glaser and Glaser 1990; Brown et al. 2005; Ascoli 2006) and are thus approximations. Future development of unbiased probes for quantification of length and surface area (Howard and Reed 1998) at the single-cell level will provide better estimates of synaptic density. However, the approximations can still be considered valuable for comparisons between different dendritic domains of an individual neuron, and with data from other neurons obtained using the same method.

The protocol requires about 12 weeks to complete, most of the time being allocated to processing for ultrastructural analysis (Table 2). An alternative to reduce processing time and that merits attention would be the use of epifluorescent or confocal microscopy for the identification of putative synapses (Wouterlood et al. 2002, 2003; Henny and Jones 2006, 2008; Jakobs et al. 2008) using immunohistochemistry against neurotransmitter-specific pre-synaptic and post-synaptic markers (Kornau et al. 1995; Chaudhry et al. 1998; Sassoe-Pognetto et al. 2000; Fremeau et al. 2001; Henny et al. 2012). This would also allow a significant increase in the fraction of tissue sampled, an improvement in CE values for accuracy of estimates, and a better resolution for single neuronal compartments (see above). These approaches and tools could allow a reasonable compromise between definitive verification of synaptic identity, time that must be invested in ultrastructural analyses, and accuracy.

**Table 2:** Timing of procedure

Procedure	Steps^a^	Duration (days)
Juxtacellular labeling of single neuron	1	1–2
Tissue processing for microscopic analyses	2–3	3–6
Digital tracing of labeled neuron	4–11	4–14
Set-up for stereological sampling	12–15	3–4
Re-embedding of tissue	16–19	3–5
Ultrathin sectioning	20–22	15–20
Acquisition of electron micrographs	23–24	10–15
Counting of synapses	25–29	2–3
Co-registration and further analysis of data	30–34	4–6
Total days	1–34	45–75
Total working weeks (5 days)	1–34	9–15 (weeks)

^a^From step by step protocol 2, Supplementary Methods

## Electronic supplementary material

Below is the link to the electronic supplementary material.

Supplementary material 1 (DOC 473 kb)

## References

[CR1] Ascoli GA (2006). Mobilizing the base of neuroscience data: the case of neuronal morphologies. Nat Rev Neurosci.

[CR2] Avendano C (2006) Stereology of neural connections: an overview. In: Zaborszky L, Wouterlood FG, Lanciego JL (eds) Neuroanatomical tract-tracing 3. Springer, US, pp 477–529

[CR3] Bolam JP (1992). Experimental neuroanatomy: a practical approach. The practical approach series.

[CR4] Brown KM, Donohue DE, D’Alessandro G, Ascoli GA (2005). A cross-platform freeware tool for digital reconstruction of neuronal arborizations from image stacks. Neuroinformatics.

[CR5] Brown MT, Henny P, Bolam JP, Magill PJ (2009). Activity of neurochemically heterogeneous dopaminergic neurons in the substantia nigra during spontaneous and driven changes in brain state. J Neurosci.

[CR6] Cajal SR (1899) Inducciones fisiológicas de la morfología y conecciones de las neuronas. In: Textura del Sistema Nervioso del Hombre y de los Vertebrados, vol Tomo I. Imprenta y Librería de Nicolás Moya, Madrid, pp 77–111

[CR7] Chaudhry FA, Reimer RJ, Bellocchio EE, Danbolt NC, Osen KK, Edwards RH, Storm-Mathisen J (1998). The vesicular GABA transporter, VGAT, localizes to synaptic vesicles in sets of glycinergic as well as GABAergic neurons. J Neurosci.

[CR8] Coggeshall RE, Lekan HA (1996). Methods for determining numbers of cells and synapses: a case for more uniform standards of review. J Comp Neurol.

[CR9] DeFelipe J (2010). From the connectome to the synaptome: an epic love story. Science.

[CR10] Denk W, Horstmann H (2004). Serial block-face scanning electron microscopy to reconstruct three-dimensional tissue nanostructure. PLoS Biol.

[CR11] Duque A, Zaborszky L (2006) Juxtacellular labeling of individual neurons in vivo: from electrophysiology to synaptology. In: Zaborszky L, Wouterlood FG, Lanciego JL (eds) Neuroanatomical tract-tracing 3. Springer, US, pp 197–236

[CR12] Faunes M, Fernandez S, Gutierrez-Ibanez C, Iwaniuk AN, Wylie DR, Mpodozis J, Karten HJ, Marin G (2012) Laminar segregation of GABAergic neurons in the avian nucleus isthmi pars magnocelluraris: a retrograde tracer and comparative study. J Comp Neurol. doi:10.1002/cne.23253 10.1002/cne.23253 23124899

[CR13] Fremeau RT, Troyer MD, Pahner I, Nygaard GO, Tran CH, Reimer RJ, Bellocchio EE, Fortin D, Storm-Mathisen J, Edwards RH (2001). The expression of vesicular glutamate transporters defines two classes of excitatory synapse. Neuron.

[CR14] Glaser JR, Glaser EM (1990). Neuron imaging with Neurolucida—a PC-based system for image combining microscopy. Comput Med Imaging Graph.

[CR15] Grillner S, Deliagina T, Ekeberg O, el Manira A, Hill RH, Lansner A, Orlovsky GN, Wallen P (1995). Neural networks that co-ordinate locomotion and body orientation in lamprey. Trends Neurosci.

[CR16] Gritti I, Henny P, Galloni F, Mainville L, Mariotti M, Jones BE (2006). Stereological estimates of the basal forebrain cell population in the rat, including neurons containing choline acetyltransferase, glutamic acid decarboxylase or phosphate-activated glutaminase and colocalizing vesicular glutamate transporters. Neuroscience.

[CR17] Gulyas AI, Megias M, Emri Z, Freund TF (1999). Total number and ratio of excitatory and inhibitory synapses converging onto single interneurons of different types in the CA1 area of the rat hippocampus. J Neurosci.

[CR18] Hausser M, Spruston N, Stuart GJ (2000). Diversity and dynamics of dendritic signaling. Science.

[CR19] Henny P, Jones BE (2006). Innervation of orexin/hypocretin neurons by GABAergic, glutamatergic or cholinergic basal forebrain terminals evidenced by immunostaining for presynaptic vesicular transporter and postsynaptic scaffolding proteins. J Comp Neurol.

[CR20] Henny P, Jones BE (2008). Projections from basal forebrain to prefrontal cortex comprise cholinergic, GABAergic and glutamatergic inputs to pyramidal cells or interneurons. Eur J Neurosci.

[CR21] Henny P, Brown MT, Northrop A, Faunes M, Ungless MA, Magill PJ, Bolam JP (2012). Structural correlates of heterogeneous in vivo activity of midbrain dopaminergic neurons. Nat Neurosci.

[CR22] Howard CV, Reed MG (1998). Unbiased stereology. Three-dimensional measurement in microscopy. Microscopy; cell and developmental biology.

[CR23] Izhikevich EM, Edelman GM (2008). Large-scale model of mammalian thalamocortical systems. Proc Natl Acad Sci USA.

[CR24] Jakobs TC, Koizumi A, Masland RH (2008). The spatial distribution of glutamatergic inputs to dendrites of retinal ganglion cells. J Comp Neurol.

[CR25] Jarsky T, Roxin A, Kath WL, Spruston N (2005). Conditional dendritic spike propagation following distal synaptic activation of hippocampal CA1 pyramidal neurons. Nat Neurosci.

[CR26] Javier RM, Kreitzer AC (2012). Dendritic architecture: form and function. Nat Neurosci.

[CR27] Katz Y, Menon V, Nicholson DA, Geinisman Y, Kath WL, Spruston N (2009). Synapse distribution suggests a two-stage model of dendritic integration in CA1 pyramidal neurons. Neuron.

[CR28] Klausberger T, Somogyi P (2008). Neuronal diversity and temporal dynamics: the unity of hippocampal circuit operations. Science.

[CR29] Kornau HC, Schenker LT, Kennedy MB, Seeburg PH (1995). Domain interaction between NMDA receptor subunits and the postsynaptic density protein PSD-95. Science.

[CR30] Megias M, Emri Z, Freund TF, Gulyas AI (2001). Total number and distribution of inhibitory and excitatory synapses on hippocampal CA1 pyramidal cells. Neuroscience.

[CR31] Miki T, Fukui Y, Itoh M, Hisano S, Xie Q, Takeuchi Y (1997). Estimation of the numerical densities of neurons and synapses in cerebral cortex. Brain Res Brain Res Protoc.

[CR32] Pakkenberg B, Gundersen HJ (1997). Neocortical neuron number in humans: effect of sex and age. J Comp Neurol.

[CR33] Petreanu L, Mao T, Sternson SM, Svoboda K (2009). The subcellular organization of neocortical excitatory connections. Nature.

[CR34] Pinault D (1996). A novel single-cell staining procedure performed in vivo under electrophysiological control: morpho-functional features of juxtacellularly labeled thalamic cells and other central neurons with biocytin or neurobiotin. J Neurosci Methods.

[CR35] Saper CB (1996). Any way you cut it: a new journal policy for the use of unbiased counting methods. J Comp Neurol.

[CR36] Sassoe-Pognetto M, Panzanelli P, Sieghart W, Fritschy JM (2000). Colocalization of multiple GABA(A) receptor subtypes with gephyrin at postsynaptic sites. J Comp Neurol.

[CR37] Segev I, London M (2000). Untangling dendrites with quantitative models. Science.

[CR38] Spruston N (2008). Pyramidal neurons: dendritic structure and synaptic integration. Nat Rev Neurosci.

[CR39] Sterio DC (1984). The unbiased estimation of number and sizes of arbitrary particles using the disector. J Microsc.

[CR40] Swanson LW (2007). Quest for the basic plan of nervous system circuitry. Brain Res Rev.

[CR41] Tang Y, Nyengaard JR, De Groot DM, Gundersen HJ (2001). Total regional and global number of synapses in the human brain neocortex. Synapse.

[CR42] Vanhecke D, Studer D, Ochs M (2007). Stereology meets electron tomography: towards quantitative 3D electron microscopy. J Struct Biol.

[CR43] West MJ (1999). Stereological methods for estimating the total number of neurons and synapses: issues of precision and bias. Trends Neurosci.

[CR44] West MJ, Slomianka L, Gundersen HJ (1991). Unbiased stereological estimation of the total number of neurons in the subdivisions of the rat hippocampus using the optical fractionator. Anat Rec.

[CR45] Wouterlood FG, van Haeften T, Blijleven N, Perez-Templado P, Perez-Templado H (2002). Double-label confocal laser-scanning microscopy, image restoration, and real-time three-dimensional reconstruction to study axons in the central nervous system and their contacts with target neurons. Appl Immunohistochem Mol Morphol.

[CR46] Wouterlood FG, Bockers T, Witter MP (2003). Synaptic contacts between identified neurons visualized in the confocal laser scanning microscope. Neuroanatomical tracing combined with immunofluorescence detection of post-synaptic density proteins and target neuron-markers. J Neurosci Methods.

