# Supplementary material for: Stereological and ultrastructural quantification of the afferent synaptome of individual neurons
Source: Brain Struct Funct. 2013 Mar 12;219(2):631–40. doi: 10.1007/s00429-013-0523-9 (PMC3933745; doi:10.1007/s00429-013-0523-9)
Supplement: Supplementary file 1 — Supplementary material 1 (DOC 473 kb) [file 429_2013_523_MOESM1_ESM.doc]

***Brain Structure and Function***

**SUPPLEMENTARY METHODS**

**Stereological and ultrastructural quantification of the afferent synaptome of individual neurons.**

Pablo Henny^1, 2, 3^, Matthew T. C. Brown^1,†^, Benjamin R. Micklem^1^, Peter J. Magill^1,4^ and J. Paul Bolam^1,4^

^1^MRC Anatomical Neuropharmacology Unit, Department of Pharmacology, University of Oxford, Mansfield Road, Oxford OX1 3TH, United Kingdom. ^2^Laboratory of Neuroanatomy, Departamento de Anatomía Normal, Escuela de Medicina, and ^3^Centro Interdisciplinario de Neurociencia, Pontificia Universidad Católica de Chile, Lira 44, Santiago, Chile; ^4^Oxford Parkinson’s Disease Centre, University of Oxford, United Kingdom.

†Current address: Department of Bioengineering, Imperial College, London, UK.

Corresponding author: Pablo Henny, E-mail: phenny@med.puc.cl, Phone: +56 2 354 3076

**Supplementary Methods I: *In vivo* labeling and tissue processing for fluorescence, light and electron microscopy**

Procedures described in this section do not differ substantially from standard electrophysiological, histochemical or immunohistochemical protocols. Below, we provide a brief summary referring the reader to references.

***Juxtacellular labeling:*** Refer to literature for more detailed descriptions of the juxtacellular recording/labeling technique . Typically, the animal is anesthetized and its head is fixed in a stereotaxic frame. A glass electrode (10–25 MΩ *in situ*, 1.0-1.5 µm tip), filled with 0.5 M NaCl solution containing 1–2% w/v of the tracer, neurobiotin ([Vector Laboratories](http://www.vectorlabs.com/uk/default.aspx)), is slowly advanced into the brain region of interest, where action potentials are amplified, filtered and recorded (Supplementary Fig. 1). The recorded neuron is labeled using a pulsed application of small positive currents (typically < 10 nA) delivered in the juxtamembranous position (Supplementary Fig. 1). The electrode is then retracted, and time allowed for anterograde transport of the tracer throughout the neuron (usually 2-15 h). The animal is then perfused through the ascending aorta with fixative and the brain removed for anatomical analyses.

***Neurochemical characterization of labeled single neuron (optional, see steps 5-11 in Step by step protocol 1).*** After the fixed brain is sectioned and collected in series, the tissue sections containing the brain region in which electrophysiological recording and cell labeling with biotinylated tracer (neurobiotin) are performed are isolated. These sections are then incubated in fluorophore-conjugated streptavidin, which binds to the tracer, and then washed and mounted and examined in an epifluorescent or confocal microscope . After the tissue sections containing the cell body and adjacent primary dendrites are found, they are unmounted, washed and incubated in a primary antibody (raised against the neurochemical or other molecular marker of interest) overnight at room temperature. Avoid the use of Triton or other permeabilizing reagents that will compromise tissue ultrastructure. The following day, the sections are incubated in fluorophore-conjugated secondary antibodies, and washed and mounted for examination of neurobiotin/antigen colocalization by epifluorescence (Supplementary Fig. 1). After verification of the neurochemical/molecular phenotype of the neurobiotin-labeled neuron, the sections are washed and placed together with the rest of the sections for further processing.

***Processing for light and electron microscopic visualization of cell body and dendrites (required, see steps 12-15 in Step by step protocol 1):*** All tissue sections containing neurobiotin-labeled processes are incubated in a cryoprotectant solution overnight and then freeze-thawed to enhance the penetration of the immunoreagents. These sections are then incubated overnight in an avidin-biotin-peroxidase (ABC) solution. The sections are then washed and incubated in diaminobenzidine (DAB) in the presence of hydrogen peroxide and nickel ions to reveal the neuron by a permanent, dark blue/black reaction product that is electron dense. Sections are washed, post-fixed in osmium, dehydrated and embedded in resin (Supplementary Fig. 1) .

***Labeling of presynaptic terminals (optional, see step 16 and 25 to 30 in Step by step protocol 1):*** After freeze-thawing (and before post-fixation, dehydration and resin embedding), the sections are incubated overnight in primary antibodies raised against presynaptic axon terminal markers such as vesicular transporters . Then the neurobiotin-labeled cell and dendrites is revealed as explained above by incubation in the ABC solution and then Ni-DAB plus H_2_O_2_. The presynaptic markers are then revealed using a peroxidase-anti-peroxidase step: In brief, the sections are incubated for 4 h in an unconjugated secondary antibody, washed, and then incubated for 4 h in a peroxidase-anti-peroxidase complex (made in the same species as the primary antibody); presynaptic terminals are revealed by carrying out a DAB reaction without nickel to give brown, electron-dense product (not shown, see Henny et al. ). Sections are then post-fixed in osmium, dehydrated and embedded in resin .


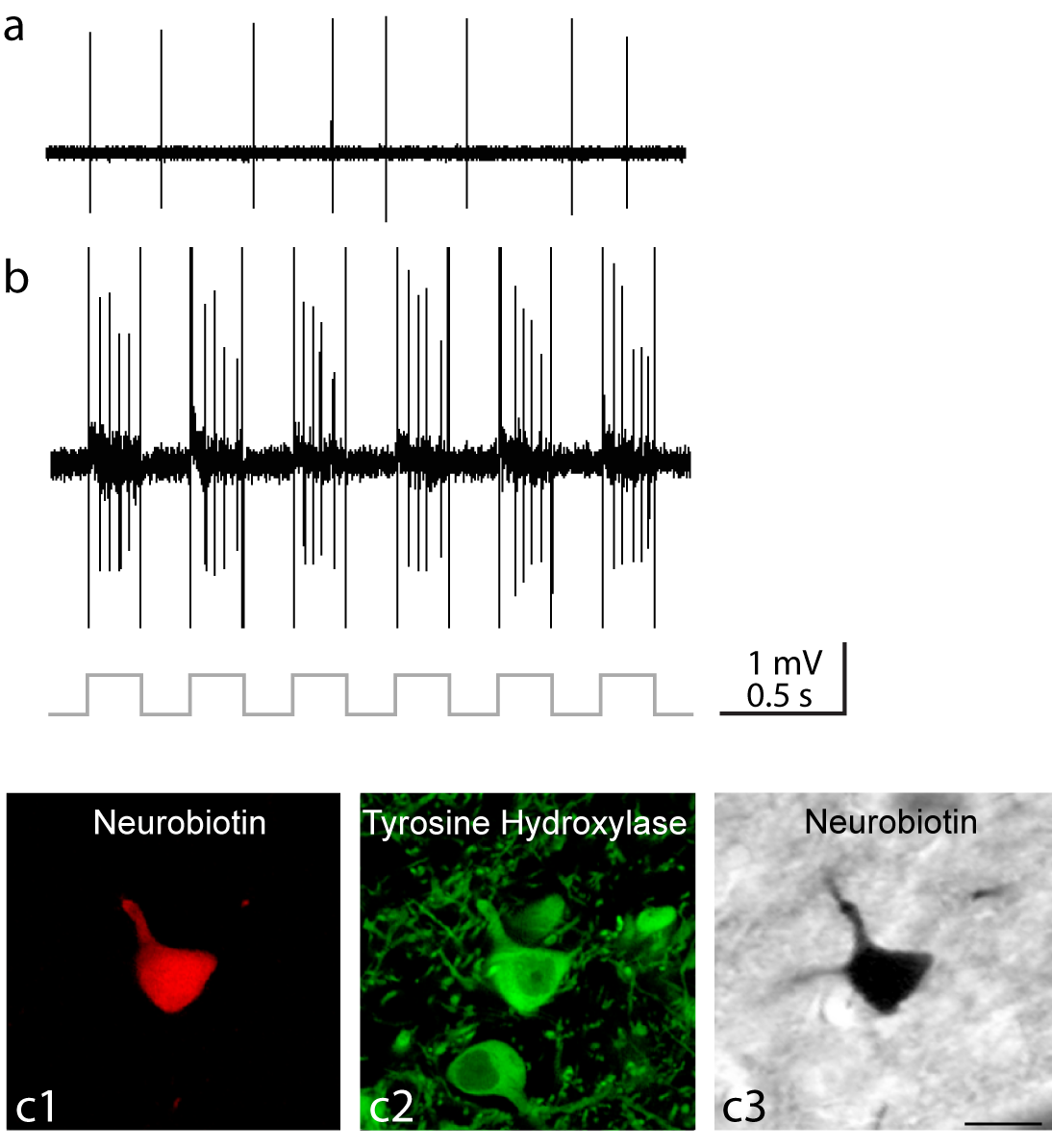


**Supplementary Fig. 1 In vivo labeling and histological processing for fluorescence, light and electron microscopy. a**: Spontaneous single-unit activity recorded in the substantia nigra in an anesthetized adult rat. Note the slow, regular discharges typical of a midbrain dopaminergic neuron. **b**: Same neuron during the application of small positive current pulses (~3 nA, in gray) in a juxtacellular configuration. Unit activity is increased during periods of current application, indicating effective juxtacellular labeling. **c1.** Single-plane confocal micrograph of a neurobiotin-labeled cell body in the substantia nigra of the rat, as revealed using Cy3-streptavidin (red). **c2.** This neuron was also immunolabeled for tyrosine hydroxylase, as revealed with AlexaFluor-488 conjugated secondary antibodies (in green), establishing its neurochemical phenotype as dopaminergic. **c3.** The same neuron is seen after performing a peroxidase reaction to permanently reveal the neurobiotin with Ni-DAB, which allows tracing in the light microscope (see Fig. 2a) and ultrastructural analysis in the electron microscope (see Fig. 3).

***Step by step protocol 1***

*Localization of cell body and first assessment of tracer labeling*

1 Incubate all free-floating brain sections in Cy3-conjugated streptavidin (Zymed) at 1:1000 dilution in PBS for 3 h at room temperature. NOTE: Do not use Triton or any other permeabilizing reagent or procedure that may compromise the tissue ultrastructure.

2 Wash sections 3 x 10 min in PBS, flat mount on microscopic slides with Vectashield ([Vector Laboratories](http://www.vectorlabs.com/uk/default.aspx)) and apply coverslip.

3 Using an epifluorescence microscope, locate the cell body and assess overall quality of neurobiotin/streptavidin labeling (Supplementary Fig. 1c). The user must have confidence that the soma and dendrites are completely labeled.

4 Unmount sections, rinse in PBS 3 x 10 min and leave in PBS. NOTE: the sections can be left at 4°C for 1 to 3 days, or for longer periods in PBS containing 0.05% sodium azide ([Sigma](http://www.sigmaaldrich.com/)). After azide, wash 6 x 10 min in PBS.

*Neurochemical characterization (Optional step: go to step 12 if not carried out)*

Presence of a molecular marker in the neurobiotin-labeled neuron can be assessed using standard immunohistochemical procedures .

5 Incubate sections in PBS containing normal serum (e.g. normal donkey serum, NDS, [Jackson Immunoresearch](http://www.jacksonimmuno.com/)) for 30 min.

6 Incubate sections in primary antibody or antibodies overnight at room temperature. NOTE: Do not use Triton or any other permeabilizing procedure that may compromise ultrastructure.

7 Wash in PBS, 3 x 10 min.

8 Incubate sections in fluorophore (e.g. AlexaFluor-488, [Invitrogen](http://www.invitrogen.com/)) -conjugated secondary antibody for 3-12 h at room temperature.

9 Wash in PBS, 3 x 10 min and flat mount using Vectashield medium.

10 Examine sections by epifluorescence or confocal microscopy and acquire images (see Supplementary Fig. 1b). NOTE: because no permeabilizing procedure is used at this stage, fluorescent staining will likely be restricted to tissue at or near the surfaces of the 50 µm sections. It is in this surface region where co-localization of neurobiotin/streptavidin and antibodies should be judged. If antibody staining is poor, perform the immunohistochemical step after freeze-thawing (see below steps 12-15).

11 Unmount and wash sections in PBS for 3 x 10 min. NOTE: Sections can be left at 4°C for one to three days, or for longer periods in PBS containing 0.05% sodium azide. After azide, wash 6 x 10 min in PBS.

*Processing for light and electron microscopy*

12 Incubate sections in cryoprotectant solution for at least 3 h or overnight at room temperature, ensuring the sections sink.

13 Pour ~200 ml of liquid nitrogen into a 500 ml plastic beaker. Pour ~100 ml of isopentane in a 250 ml plastic beaker and then place it inside the larger beaker to cool it down.

14 Place tissue sections in mesh wells and remove excess cryoprotectant solution by placing wells on absorbent paper.

15 Place a mesh well in the cold isopentane for ~8 s, then quickly remove the isopentane-filled beaker and place the same mesh directly into the liquid nitrogen for ~4 s. Leave mesh well on bench top so that sections thaw (regain translucency). Quickly place wells back in cryoprotectant, gently remove sections and place them in PBS again. Wash 3 x 10 in PBS.

*Labeling of presynaptic terminals (Optional step: go to step 18 to continue the processing of tracer-labeled single cell if not carried out,)*

For neurochemical characterization of presynaptic terminals at the ultrastructural level, an additional immunohistochemical labeling step can be carried out using a peroxidase procedure. NOTE: It is important that the targeted protein or marker exclusively labels axon terminals in the region of interest and not cell bodies or dendrites because the latter may preclude unambiguous identification of the neurobiotin-labeled neuron.

16 Incubate sections in primary antibody (selecting a host species different from that used for molecular characterization of cell body and dendrites in steps 5-11) against molecular markers of presynaptic terminals overnight at room temperature.

17 Wash sections in PBS for 3 x 10 min. NOTE: Do not add the secondary antibodies yet.

18 Prepare an avidin-biotin peroxidase (ABC) solution in PBS according to manufacturer guidance (ABC or ABC Elite, [Vector Laboratories](http://www.vectorlabs.com/uk/default.aspx)).

19 Incubate all sections in ABC for at least 3 h (preferably overnight).

20 Incubate sections in 0.05 M Tris buffer ([Sigma](http://www.sigmaaldrich.com/)), pH 8.0, for 10 min.

21 Transfer sections to Tris buffer containing nickel (nickel ammonium sulphate, 0.5% w/v, [Sigma](http://www.sigmaaldrich.com/)) and diaminobenzidine tetrahydrochloride (DAB, 0.025% w/v; [Sigma](http://www.sigmaaldrich.com/)) and leave for further 10 min.

22 Separate section containing the cell body to a new well with the same solution, add H_2_O_2_ ([Sigma](http://www.sigmaaldrich.com/)) up to a final concentration of 0.002% w/v, and incubate for 2 - 20 min.

23 Establish optimal time of incubation; check Ni-DAB staining intensity every 2 min by stopping the reaction (washing in Tris buffer), mounting, covering and observing in a light microscope. Though somewhat blurry, cell body should be visualized by the blue/black precipitate.

24 After optimization, add H_2_O_2_ to the rest of the sections and incubate for the established time. Wash sections in PBS 6 x 10 min at end of procedure.

*Follow steps 25 to 30 if performing additional staining for presynaptic markers. Otherwise, skip to step 31.*

25 Incubate sections in the unconjugated secondary antibody, made against the host species of primary antibodies raised against presynaptic terminal markers, at a dilution of 1:100 for 2 h.

26 Incubate sections in a peroxidase-conjugated tertiary antibody, made in the same host species as the primary antibodies (PAP, [Jackson Immunoresearch](http://www.jacksonimmuno.com/)), at a dilution of 1:400 for 4 h.

27 Incubate sections in 0.05M Tris buffer, pH 7.6, for 10 min.

28 Incubate sections in Tris buffer containing DAB (without nickel) for 10 min.

29 Choose a single section, add H_2_O_2_ to a final concentration of 0.002% w/v and, as explained in step 23, optimize incubation time to obtain robust visualization of presynaptic markers with a brown precipitate.

30 After optimization, repeat for all sections. Then wash sections in PBS 6 x 10 min. NOTE: Sections can be left at 4°C for one to three days, or for longer periods in PBS containing 0.05% sodium azide. After azide, wash well for 6 x 10 min in PBS.

31 Equilibrate all sections with 0.1 M phosphate buffer, pH 7.4 (PB).

32 Place sections in small glass Petri dishes, pipette off the PB and ensure that the sections lie flat in the dish.

33 Carefully immerse sections in a small volume of 1% osmium tetroxide ([Oxkem](http://www.oxkem.com/)) in PB and cover. Ensure that the sections do not float up or curl in the solution.

34 Leave in osmium tetroxide solution for 25 min. NOTE: If doing double labeling using DAB and Ni-DAB, treat sections with 1% osmium ([Sigma](http://www.sigmaaldrich.com/)) in 0.1M PB pH 7.4 containing 5% Beta-D- glucose for up to 60 min.

35 During incubation in osmium, prepare one aluminum foil boat for each Petri dish containing sections. Use a dry Petri dish to shape the foil.

36 Also prepare Durcupan resin ([Fluka](http://www.sigmaaldrich.com/)), mixing the components A:B:C:D in the ratios 10:10:0.3:0.2 (by weight), and leave aside.

37 Pipette off the osmium solution into a specific waste osmium bottle.

38 Wash sections 3 x 15 min in PB.

39 Prepare 1% w/v uranyl acetate ([TAAB](http://www.taab.co.uk/)) in 70% v/v ethanol.

40 In the same Petri dishes, treat sections sequentially with the following:

I. 50% ethanol for 15 min. NOTE: Use a large volume to ensure that all the phosphate is removed because any remaining phosphate may react with the uranyl acetate in the following step to produce an electron dense precipitate in the tissue.

II. 70% ethanol containing 1% uranyl acetate for 30 min. NOTE: Filter uranyl acetate before applying to sections.

III. 95% ethanol for 15 min.

IV. 100% ethanol for 10-15 min.

V. Dry absolute ethanol for 10-15 min.

VI. Two changes of propylene oxide for 10-15 min each. NOTE: Sections will ripple if too much propylene oxide is removed. Ensure you leave enough propylene oxide in the Petri dishes during the changes so that it does not evaporate entirely.

41 Pour Durcupan resin into the aluminum foil boats.

42 Using tweezers, rapidly transfer the sections from the last propylene oxide wash to the aluminum boats. NOTE: Ensure that the sections do not dry out at this stage and that they are completely submerged in the resin.

43 Leave sections in Durcupan resin overnight in a fume hood.

44 The following day, gently warm the boats containing the sections and the resin on a hot plate and transfer the sections, one at a time, to skin-greased glass microscope slides. Allow them to settle for a few minutes. NOTE: Carefully apply a thin layer of grease to slides by running them against your forehead. This will aid the removal of coverslips and tissue blocks during the re-embedding procedure at a later date.

45 Examine the slides under a dissection microscope to ensure that tissue sections, or fragments of sections, are not overlying each other. NOTE: The sections or fragments can be maneuvered about the slide with cocktail sticks or a paint brush.

46 Place a coverslip on top of sections. Gently and carefully, grease coverslips from your skin and place coverslip over sections. Allow sections and resin to settle and then press down gently to remove all air bubbles and any excess resin. NOTE: The amount of resin should be sufficient to cover the sections and spread to the edge of the coverslip by capillary action but not to emerge from the sides when the coverslip is pressed. Excess resin can be removed by absorbing it onto filter paper, or removing it with cotton buds. Conversely, extra resin can be added by placing drops along the edge of the coverslip.

47 Place the slides in containers (shallow cardboard trays lined with aluminum foil) and polymerize the resin by heating in an oven at 60°C for 48 h.

48 Assess overall quality of resin-embedded tissue and staining at the light microscopic level (Fig. 2 and Supplementary Fig. 1c).

**Supplementary Methods II: Protocol for stereological and ultrastructural quantification of synaptic inputs and their somatodendritic distribution.**

***Supplies and Equipment***

*Information on Supplies and Equipment is only provided for those used in steps 4-34. For details about materials referred to in steps 1-3, see Supplementary Methods I and the original publications as cited.*

*Re-embedding*

Cyanoacrylate adhesive (Super Glue, [Loctite](http://www.halfords.com/))

Single-edged carbon-steel razor blade ([Agar Scientific UK](http://www.agarscientific.com/catalogue/action_catalogue.asp?sat=2&saa=4&path=(S|1||razor blade|AND||AND|||)(D||5|T585|0|S)))

Double-edged razor blade [(Wilkinson](http://www.needblades.co.uk/classic-double-edge-10-blade-pack-28-p.asp))

Wooden cocktail sticks

Scalpel blade ([Swann-Morton](http://www.swann-morton.com/product/17.php), size 10A)

Pioloform ([Agar](http://www.agarscientific.com/catalogue/action_catalogue.asp?sat=2&saa=3), R1275)

Single-slot copper grids ([Agar](http://www.agarscientific.com/catalogue/action_catalogue.asp?sat=2&saa=3), G2500C)

Fine metal tweezers ([Agar](http://www.agarscientific.com/catalogue/action_catalogue.asp?sat=2&saa=3), T5297)

Dissecting microscope (Leica Microsystems, [WILD M3B](http://eolsurplus.com/images/WildM3B.jpg))

Ultramicrotome (Leica Microsystems, [EM UC6](http://www.leica-microsystems.com/products/electron-microscope-sample-preparation/biological-specimens/low-temperature-techniques/ultramicrotomy/details/product/leica-em-uc6/)).

Diamond knife ([Diatome knives](http://www.diatomeknives.com/), MS3715)

Reichert knifemaker (Leica Microsystems)

Glass strip knife blanks (Leica Microsystems)

Anti-static gun ([Agar](http://www.agarscientific.com/catalogue/action_catalogue.asp?sat=2&saa=3), G375)

*Serial ultramicrograph acquisition*

Transmission electron microscope (Philips CM100)

Digital camera (UltraScan 1000 CCD camera, [Gatan](http://www.gatan.com/))

DigitalMicrograph software ([Gatan](http://www.gatan.com/))

*3D digital reconstruction and stereological analysis*

Light microscope ([Eclipse 80i, Nikon](http://www.microscopyu.com/)) equipped with 2x to 100x (1.4 NA) range of objectives

High-resolution digital camera Motorized x, y stage ([LUDL Electronic Products](http://www.ludl.com/))

Stepper-motor focus drive ([LUDL Electronic Products](http://www.ludl.com/))

z-axis linear encoder (MT12, [Heidenhain](http://www.heidenhain.co.uk/))

3-axis stage controller (MAC 5000, [LUDL Electronic Products](http://www.ludl.com/))

Lucivid CRT monitor for microscope’s drawing tube ([MBF Bioscience](http://www.mbfbioscience.com/))

Dedicated PC and PC monitor

Neurolucida software (v8.0, [MBF Bioscience)](http://www.mbfbioscience.com/)

Stereo Investigator software (v8.0, [MBF Bioscience](http://www.mbfbioscience.com/))

Neurolucida Explorer software (v4.70.3 [MBF Bioscience](http://www.mbfbioscience.com/))

***Step by step protocol 2***

*Labeling and identification of a single neuron*

1 **Labeling**: Record electrical activity and label a single neuron with a neuronal tracer (in this case, neurobiotin) using the juxtacellular technique in the animal (in this case, the anesthetized rat) (see Supplementary methods I). After appropriate survival time, perfuse-fix the animal and remove the brain.

*Tissue processing for light and electron microscopic analyses*

2 **Collecting tissue sections**: Mount brain tissue on cutting stage of a vibrating microtome and start collecting ‘free floating’ serial sections at 50 µm far away enough from the tracer-labeled cell to ensure that the entire somato-dendritic compartment is collected. To ensure random sampling in the z-axis (defined here as the axis perpendicular to the plane of sectioning), advance cutting stage a random distance (r) immediately before section collection starts (for 50 µm thick serial sections, choose an r between 1 and 50 µm. Visit <http://www.random.org/>).

3 **Tissue processing**: Process the tissue for localization of cell body of tracer-labeled neuron, optional testing of neurochemical identity, permanent visualization of somato-dendritic architecture by a peroxidase method, optional permanent visualization of presynaptic axon terminal markers, and embedding in an electron microscope resin on microscope slides. See description and options of tissue processing in Step by step protocol 1.

*Digital tracing of labeled neuron*

4 **Put sections in order**: Examine sections at low magnification to confirm the order in which they should be mounted on slides for further analysis.

5 **Set the serial section manager**: Using the serial section manager (SSM) function of the Neurolucida software, create a SSM section for the tissue section containing the cell body, which is the first to be traced. Give this first SSM section a name (usually a name specifying the slide and position of the tissue section), a depth value for the top surface of the section (usually zero µm) as well as the constant block advance that was used when cutting the brain on the vibrating microtome (50 µm).

6 **Trace relevant contours**: Digitally trace contours of the region of interest using a low-magnification objective. Ensure all the labeled neuronal processes are encompassed in the contour trace (e.g., if tracing a dopaminergic neuron, define the contours of both pars compacta and pars reticulata into which the dendrites extend).

7 **Trace the cell body**: Start tracing the soma under oil immersion using a 100x objective. In order to obtain a close approximation of its three-dimensional shape and size, use the contour drawing mode and draw contours separated by 1 or 2 µm in the z-axis.

8 **Trace dendrites in the section**: Trace all fragments of dendrite and axon (100x objective) using the neuron drawing mode. When reaching the surface of the tissue section, name the fragment endings accordingly (e.g. ‘H’ for high ending on top of surface; ‘L’ for low ending on bottom of section; ‘M’ for endings in the middle of the section) (Fig. 1b, c). Once finished, be sure to scan the entire region to trace dendritic fragments not traced already (usually fragments unconnected to the main dendritic shaft or cell body in that brain section).

9 **Trace landmarks**: Trace some landmarks within the tissue, e.g. blood vessels, to help in the later aligning and splicing of the tissue sections.

10 **Align the next tissue section and set up a new SSM section**: Align an adjacent brain section with first SSM section at low magnification. Then create a new SSM section as explained in step 5 and repeat steps 6 to 9. NOTE: Dendritic fragments traced in the new SSM section must not be spliced to dendritic fragments in previous SSM sections at this stage.

11 **Complete tracing and align dendritic fragments:** Move to next adjacent section and repeat steps 5 to 10 until all the dendritic fragments in all brain sections have been found and traced. Manually align dendritic fragments (Fig. 5a). Save version of file (referred to as the ‘unspliced’ reconstruction).

*Set up for stereological sampling of synapses*

12 **Photograph and number all dendritic fragment “high endings”**: Starting from one of the two tissue sections most distal to the soma, re-visit all labeled dendritic fragments in all tissue sections of interest (100x objective). Focus at the top of each section, and acquire an image of the dendritic fragment high endings (Fig. 2a). Number each high ending. Print out the image with and without the respective tracing (Fig. 2a). Also acquire images at medium (40x or 20x) and low (10x or 2x) magnification, and create a digital file (e.g. PDF) of the tracing overlaid on the captured images (Fig. 2b).

13 **Open reconstruction file in Stereo Investigator**: Open the unspliced reconstruction of the neuron in Stereo Investigator and save a new version of it. Open the SSM function to see all SSM sections as created previously in Neurolucida.

14 **Define counting frame and prepare stereological probe**: Define a counting frame of 100 µm x 100 µm (since we are sampling 100% of the area, these dimensions are not particularly critical). Choose the Optical Fractionator probe in Stereo Investigator and define a counting grid (*XY Placement of Counting Frame*) equal to the counting frame, in order to sample 100% of the area. In the Disector Probe Settings (within the Optical Fractionator Probe) set the Guard Zone at a fixed distance of 0.05 µm (see below). Define an Optical Disector Height of 0.5 µm (which corresponds to the thickness of tissue that will be sampled at the electron microscopic level, see below). Finally, enter the Mounted Section Thickness, which is the thickness of sections as measured after processing (46 µm for a section originally cut with a 50 µm block advance, which corresponds to a shrinkage in thickness of ~8%, as measured in this and previous material in our laboratory ).

15 **Checking step**: Ensure that all the dendritic fragment high endings have been imaged (step 12) and adequately recorded (after the following step it will not be possible to re-visit the dendritic processes).

*Re-sectioning and re-embedding of tissue for electron microscopy*

16 **Remove coverslips**: Using a dissection microscope with variable magnification (6.4x to 40x), remove the slide coverslip by cleaning out all of the resin from above and over the edges of the coverslip using a double-edged razor blade. NOTE: Break a thin double-edged razor blade into two halves and carefully insert a half-blade between the coverslip and the underlying resin. Advance over the section to remove the coverslip. Remove any pieces of glass that remain if the coverslip breaks. NOTE: Coverslip removal is facilitated by having the sections colder than room temperature (placing them in a fridge for a few minutes beforehand is sufficient).

17 **Identify the dendritic fragment high endings to be re-sectioned and re-embedded**: Identify the precise locations of the dendritic fragment high endings to be re-sectioned with the help of the printed images (step 12, Fig. 2a, b). Aim to cut a block of tissue with as many high endings as possible contained within it, while keeping in mind that that, after trimming (see step 20), the block is not larger than ~150 µm x 600 µm (Fig. 2b). These dimensions ensure that more than 10 ultrathin sections can be placed on a single electron microscope grid (Fig. 2b, c, j).

18 **Re-section tissue:** Warm the section slightly on a hot plate. With the aid of adhesive tape, fix the slide to the base of the dissection microscope (Fig. 2d, e). Using a new half-blade taken from a double-edged razor blade, make straight cuts on both sides of the tissue region containing the dendritic fragment high endings of interest. Then turn the slide 90 degrees and make two more straight cuts, above and below the region of interest. Remove the excised piece of the tissue section (now referred to as the ‘tissue block’) with the tip of a scalpel blade (Fig. 2g). NOTE: Use a shield around the dissecting microscope to avoid losing the tissue block should it ‘flick-off’ the slide (see Fig. 2d). Leave tissue block in a safe place and cover (see Supplementary Table 1 for Troubleshooting)

19 **Re-embed tissue:** Apply a thin layer of cyanoacrylate glue to a blank resin cylinder. Place and then adhere the tissue block to the top of the resin cylinder (Fig. 2h). Number the cylinder and record the dendritic fragments contained on it. NOTE: Use a wooden cocktail stick to quickly and gently press the tissue block against the resin cylinder surface while the glue sets.

*Ultrathin sectioning of tissue for electron microscopy*

20 **Prepare block for ultrathin sectioning**: In the ultramicrotome, place the block in the base position. Using a half-blade of double-edged razor blade, trim the resin cylinder and tissue block by making oblique cuts so as to produce a trapezoid shape (Fig. 2c, im j). Check the location of the dendritic fragment high endings in a light microscope, and reduce the size of the trapezoid accordingly, while taking care not to remove any dendritic fragments. Place the block in the cutting position and remove the resin above the brain tissue using a glass knife. Approach the brain tissue surface as parallel as possible. (see Supplementary Table 1 for Troubleshooting)

21 **Collect series of ultrathin sections**: Place block in cutting position, change the glass knife to a diamond knife and start cutting ultrathin sections at the required block advance of 50 nm. Once sections start forming a ribbon, collect series of more than 10 ultrathin sections on single-slot, copper, electron microscope grids coated with pioloform . NOTE: Always cut 2-3 more ultrathin sections than needed (Fig. 2j), in case of damage to or loss of sections. After collection, briefly dry sections with a hair-dryer and store them in a grid box.

22 **Contrast sections**: Place some sodium hydroxide pellets in a Petri dish, cap it for a few minutes to allow it to absorb the CO_2_ in the dish and then place some drops of lead citrate on parafilm previously laid inside the dish . Place each grid on top of a drop of the lead citrate solution for about 5 min. Wash the grids in a stream of de-ionized water and dry with a hairdryer.

*Acquisition of electron micrographs*

23 **Identify dendritic fragment high endings**: Place the grid holding the ultrathin tissue sections in the electron microscope. Using the landmarks in the printed images taken in the light microscope (step 12), as well as those seen directly in the tissue block, find the labeled dendritic profiles in the electron microscope. (see Supplementary Table 1 for Troubleshooting)

24 **Acquiring images of ultrathin section series**: Starting with the most superficial ultrathin section of the ribbon (the one that was first cut) (Fig. 3), acquire at least 11 images (one look up, plus 10 sections of the counting region) of the dendritic fragment profiles in this and subsequent ultrathin sections, at a magnification suitable for identification of synapses. NOTE: Use a sequential nomenclature to name micrographs in a series, e.g. Block name-Grid name-Profile name-Ultrathin section number (PH177-A5-P01-UTS0001). For dendritic profile fragments that were cut tangentially, as well as fragments coming from the cell body or primary dendrites, a single micrograph may not be sufficient to include the whole profile. It may be necessary to take several electron micrographs per profile and merge them as a single image. NOTE: Use the *photomerge* function in Adobe Photoshop software to merge images from the same dendritic fragment high ending.

*Counting of synapses*

25 **Define a new lens**: In the Stereo Investigator software, create a new lens for the correct scaling of the electron micrographs (see Help: Define New Lens in Stereo Investigator)

26 **Open last sampling file**: Using this lens, open the unspliced reconstruction file that contains the Optical Fractionator probe (step 14). Open the Optical Fractionator Workflow and choose “Continue working with this subject”.

27 **Revisit sampling sites and move to traced dendritic fragment high endings:** In the workflow frame, choose the Region of Interest frame, and then select the SSM section containing the first Optical Fractionator probe. Then, select the contours where the probe was run. In the Running Options, choose “continuing the probe run”, and then click to start counting. Individually re-visit all the dendritic fragment high endings processed and imaged in the electron microscope. Move to the exact location of the traced dendritic fragment high ending.

28 **Load the respective electron** **micrograph series**: Make sure the depth value at the top of the section is correct by choosing “Reset Top of Section” on the right click menu. Then, using the Image Stack module, load the electron micrograph series for the corresponding dendritic fragment high ending. The first image of the series should be the most superficial one (closest to tissue surface). Set the distance between images to be the block advance at which the ultrathin sections were cut (0.05 µm here), and choose Focal distance Type.

29 **Count synapses**: Select a marker and count the ‘top’ of synapses as they become visible through the micrograph series. Do not count synapses whose top is outside the counting region (Fig. 1d and Fig. 3a). Repeat steps 28 and 29 for all the dendritic fragment high endings in that SSM section. Finish the Optical Fractionator probe in the SSM section and then repeat steps 28 and 29 for all SSM sections.

*Co-registration and further analysis of data*

30 **Obtain stereological results**: Open the probe list, select all sections sampled and enter 1 as the sampling interval. Obtain the number of synapses counted, the estimate of the total number of synapses received, and the coefficient of error. Save a new version of the file. NOTE: Alternative sampling schemes can be examined at this stage (Fig. 4) by, for example, selecting a reduced number of sections (Fig. 4a) or by reducing the area sampled in each section (Fig. 4b) using new probes with variable counting frame or counting grid sizes.

31 **Splice fragments of labeled neuron**: Open the file of the unspliced neuron reconstruction that was created before the sampling step (step 11, Fig. 5a, b) in Neurolucida. Save this file as a new copy (hereafter referred to as the ‘spliced’ reconstruction). Splice all somato-dendritic fragments, starting from the cell body and moving outwards. NOTE: When splicing, always select the dendritic fragment that is closest to the soma, place the mouse over its most distally traced ending, and splice to the most proximal point of the corresponding fragment in the adjacent section. Once the dendrite has been completely spliced, check that the z-value of its origin (the first point traced starting from the cell body) actually matches the z-value of the cell body.

32 **Apply shrinkage correction**: Save a new copy of the spliced reconstruction file and apply x, y and z shrinkage correction in Neurolucida to account for changes in size that occur during histological processing (shrinkage for x = 6.3%, y = 6.0%, z = 8.0%, as measured in material used in this and previous studies in our laboratory ) (Fig. 5a, b).

33 **Tag synaptic markers to spliced neuron reconstruction**: While keeping the spliced reconstruction file open in Neurolucida, open the unspliced reconstruction used for the stereological procedure (step 30) in Neurolucida Explorer. Observing the location of markers from the stereological procedure file, place markers in the spliced reconstruction file at the same locations as they were sampled. Ensure the markers in the sliced reconstruction file are attached to the respective dendrites (right-click menu: *Attach markers to object*), and check that the z-value of the markers is the same that the z-value of the point in the dendrite to which they were attached (Fig. 5c). Save this ‘tagged’ reconstruction file with a new name.

34 **Examine the distribution of synapses**: Open the tagged reconstruction file in Neurolucida Explorer and use the built-in analysis functions (*Segments analysis* in *Branched Structure Analysis*) to explore the distribution of synapses across the somato-dendritic structure (Fig. 6), including their distribution as a function of branch order (Fig. 5a, Fig. 6a), dendritic caliber (Fig. 5b, Fig. 6b) or distance from the cell body (*Sholl Analysis*) (Fig. 5c, Fig. 6c).

Supplementary Table 1. Troubleshooting

| **Step** | **Problem** | **Possible explanation** | **Solution** |
| --- | --- | --- | --- |
| **18** | Tissue block disappears during or after excision from section | Block jumped due to electrostatics | Use a anti-static ‘gun’ to reduce electrostatics on tissue and tools before excision from section  Try to find tissue block in this order: Gently remove the scalpel and carefully examine it; examine your fingers, hands and white coat on your arms; remove the bench cover shield and examine it; examine surfaces of dissection microscope, including the overlying objective lens. If not found, see alternative solution for step 23 to replace dendritic fragment high endings |
| **20** | Tissue block surface not flat | Block not flat when mounted onto resin cylinder | If tissue block surface is not flat, try to make the tissue block as small as possible (Fig. 2i). Check when the tissue surface has been reached by placing the resin cylinder in the base position of the ultramicrotome and observe block surface while tilting and rotating it. If the tissue has started to section, the surface should have a dull appearance, and it may be possible to observe cell bodies under high magnification. |
| **23** | Dendritic fragment high ending not found | Trimmed away during preparation of tissue block (Step 21) | Replace with *similar* dendritic fragment. Using spliced and shrinkage-corrected reconstruction file (step 32), identify another dendritic fragment that is similar with respect to branch order, branching points, region of interest, distance from cell body, and diameter. |
|  |  |  |  |

**REFERENCES**

Bevan MD, Booth PA, Eaton SA, Bolam JP (1998) Selective innervation of neostriatal interneurons by a subclass of neuron in the globus pallidus of the rat. J Neurosci 18 (22):9438-9452

Bolam JP (ed) (1992) Experimental Neuroanatomy: A Practical Approach. The Practical Approach Series. IRL Press, Oxford

Brown MT, Henny P, Bolam JP, Magill PJ (2009) Activity of neurochemically heterogeneous dopaminergic neurons in the substantia nigra during spontaneous and driven changes in brain state. J Neurosci 29 (9):2915-2925.

Chaudhry FA, Reimer RJ, Bellocchio EE, Danbolt NC, Osen KK, Edwards RH, Storm-Mathisen J (1998) The vesicular GABA transporter, VGAT, localizes to synaptic vesicles in sets of glycinergic as well as GABAergic neurons. J Neurosci 18 (23):9733-9750

Duque A, Zaborszky L (2006) Juxtacellular Labeling of Individual Neurons In Vivo: From Electrophysiology to Synaptology. In: Zaborszky L, Wouterlood FG, Lanciego JL (eds) Neuroanatomical Tract-Tracing 3. Springer US, pp 197-236.

Fremeau RT, Jr., Troyer MD, Pahner I, Nygaard GO, Tran CH, Reimer RJ, Bellocchio EE, Fortin D, Storm-Mathisen J, Edwards RH (2001) The expression of vesicular glutamate transporters defines two classes of excitatory synapse. Neuron 31 (2):247-260

Henny P, Brown MT, Northrop A, Faunes M, Ungless MA, Magill PJ, Bolam JP (2012) Structural correlates of heterogeneous in vivo activity of midbrain dopaminergic neurons. Nat Neurosci 15 (4):613-619.

Mena-Segovia J, Sims HM, Magill PJ, Bolam JP (2008) Cholinergic brainstem neurons modulate cortical gamma activity during slow oscillations. J Physiol 586 (Pt 12):2947-2960.

Pinault D (1996) A novel single-cell staining procedure performed in vivo under electrophysiological control: morpho-functional features of juxtacellularly labeled thalamic cells and other central neurons with biocytin or Neurobiotin. J Neurosci Methods 65 (2):113-136.

Sadek AR, Magill PJ, Bolam JP (2007) A single-cell analysis of intrinsic connectivity in the rat globus pallidus. J Neurosci 27 (24):6352-6362.

Ungless MA, Magill PJ, Bolam JP (2004) Uniform inhibition of dopamine neurons in the ventral tegmental area by aversive stimuli. Science 303 (5666):2040-2042.
